# The Hands That Save Us

**DOI:** 10.1007/s11606-025-09723-z

**Published:** 2025-07-15

**Authors:** Yamac Akgun

**Affiliations:** 1 Department of Pathology and Laboratory Medicine, Children’s Hospital Los Angeles, Los Angeles, CA USA; 2 University of Southern California Keck School of Medicine, Los Angeles, CA USA; 3 Pathology and Laboratory Medicine, University of Miami Miller School of Medicine, Miami, FL USA

**Keywords:** Blood donation, Transfusion medicine, Blood bank, Cancer

The boy was dying, and he didn’t even know it.

He was six years old, pale as the sheets beneath him, his body too weak to hold itself up. His hemoglobin had plummeted to levels barely compatible with life. His heart was beating faster, desperately trying to move what little oxygen remained in his failing blood.

We were not at his bedside. We did not see his mother’s tears. We did not hear the desperation in the doctor’s voice as they called for blood.

But we were there.

We were there in the donor center two days ago, where a woman sat in a reclining chair, squeezing a stress ball in her hand as a needle drew red from her veins. She was a nurse giving blood on her off day. She would never meet this boy, never know that her quiet act of kindness would fill the empty spaces in his body where life was slipping away.

We were there in the blood bank, where we checked, spun, crossmatched, and prepared the units that would soon be sent upstairs. We were there in the hands of the courier who carried the blood cooler to the pediatric floor, in the nurse’s steady grip as she primed the transfusion line.

We were there in the first drop that entered his veins, warm and rich with the oxygen he so desperately needed.

Blood donors do not wear capes. They do not ask for recognition. But they are the quiet army that keeps the world alive.

For every trauma patient rushed into surgery, for every child with leukemia fighting through another round of chemo, for every mother hemorrhaging after childbirth—there is someone, somewhere, rolling up their sleeve, offering life without knowing whose hands it will reach.

And behind them, we stand—the collectors, the phlebotomists, the lab techs, the apheresis specialists, the transfusion medicine doctors—making sure that every drop finds its way to where it is needed most.

No one will ever thank us by name. No patient will ever say, “You saved me.”

But we know.

And that is enough.

